# Parents' return to the hospital after the death of their children: importance of palliative care after death

**DOI:** 10.1186/cc14652

**Published:** 2015-03-16

**Authors:** G Halal, PL Lago, J Piva, M Halal

**Affiliations:** 1 Hospital de Clinicas de Porto Alegre, Brazil

## Introduction

To analyze the perception of parents regarding their return to the hospital where their children died to participate in a conversation with doctors and to analyze the feelings of parents about their participation in a study evaluating the care provided in the moments leading up to the death of children.

## Methods

A descriptive exploratory qualitative study. The study sites were the pediatric ICUs of the Hospital São Lucas and Hospital de Clinicas de Porto Alegre. Fifteen parents of children who died in the PICUs studied participated in the study. Data collection occurred in 2010 and was conducted through semistructured interviews. Data were analyzed using thematic content analysis. The research was approved by the research ethics committees of both hospitals.

## Results

The ability to return to the hospital and talk to medical assistants was considered by parents as a positive and enlightening opportunity. Parents who participated in the study understood this moment as an opportunity to be heard and demonstrated the intention to contribute with their experiences in order to improve care in the hospitals studied.

## Conclusion

We conclude that there is a need to implement measures to provide palliative care to parents after the death of their children. It is necessary to consider the possibility of providing families with follow-up meetings with the multidisciplinary team after the death of children.
